# P-1552. First-in-Human Challenge Study with Human Metapneumovirus A2: Characterization of Clinical Outcomes and Immunological Correlates

**DOI:** 10.1093/ofid/ofaf695.1732

**Published:** 2026-01-11

**Authors:** Nicolas Noulin, Alexander J Mann, Nikolay Veselinski, Mariya Kalinova, Kirsty Bradley, Julie Mori, Brandon Londt, Stephanie C Ascough, Christopher Chiu, Guy Boivin, Andrew P Catchpole

**Affiliations:** hVivo Services LTD, London, England, United Kingdom; hVIVO, London, England, United Kingdom; hVIVO, London, England, United Kingdom; hVivo Services Ltd., London, England, United Kingdom; hVIVO, London, England, United Kingdom; hVivo Services LTD, London, England, United Kingdom; hVivo, London, England, United Kingdom; Imperial College London, London, England, United Kingdom; Imperial College London, London, England, United Kingdom; Department of Microbiology-Immunology and Infectious Diseases, Laval University, Quebec City,, Quebec city, Quebec, Canada; hVivo Services Ltd., London, England, United Kingdom

## Abstract

**Background:**

Human metapneumovirus (hMPV), along with respiratory syncytial virus (RSV), is a major etiological agent of acute respiratory tract infections, particularly affecting pediatric, elderly, and immunocompromised populations. There are no approved vaccines and specific treatments against hMPV. While the RSV human challenge model (HCM) has played a critical role in antiviral and vaccine development, an analogous model for hMPV remains unavailable. This study describes the development of a novel hMPV HCM using a clinically derived A2 strain.

**Methods:**

An hMPV A2 isolate, obtained from a clinical specimen in October 2022, was propagated in qualified GMP cells and subjected to comprehensive release testing and in vitro characterization. A first-in-human pilot challenge study was conducted in twenty eight healthy adult volunteers, who were intranasally inoculated with the GMP hMPV A2 stock. Subjects were monitored over a 12-day in-unit quarantine period using symptom diaries, cold perception scores, nasopharyngeal swabs (NPS) for qPCR and viral culture, blood tests (including CBC and CRP), ECGs, spirometry, and collection of serum and PBMCs for immunological analysis. A follow-up visit was conducted on Day 28.

**Results:**

All participants completed the study without serious or significant adverse events. Volunteers developed symptoms consistent with mild upper respiratory tract infection (nasal congestion, rhinorrhea, sneezing), with symptom onset typically by Day 3 and a median duration of 7 days. Viral shedding profiles aligned with symptom kinetics. Hematologic changes and increased CRP levels were observed during acute illness. Serological analysis revealed pre- and post-challenge neutralizing antibody titres to hMPV subgroups and RSV-A/B, while paired PBMC samples were assessed for hMPV-specific T-cell responses.

**Conclusion:**

This first-in-human challenge study demonstrates the feasibility, safety, and immunological relevance of an hMPV A2 challenge model in healthy adults. The model elicited viral replication, clinical symptoms, and immune responses, supporting its utility for investigations into pathogenesis, correlates of protection, and evaluation of antiviral and vaccine candidates.

**Disclosures:**

Alexander J. Mann, BSc (Hons), hVivo Ltd.: Employee of hVivo LTD Julie Mori, PhD, hVivo Ltd.: Employee of hVivo LTD Brandon Londt, PhD, hVivo Ltd: Employee of hVivo LTD Andrew P. Catchpole, PhD, hVivo Ltd: Employee of hVivo LTD

